# Limitation of life support techniques at admission to the intensive care unit: a multicenter prospective cohort study

**DOI:** 10.1186/s40560-018-0283-y

**Published:** 2018-04-13

**Authors:** Olga Rubio, Anna Arnau, Sílvia Cano, Carles Subirà, Begoña Balerdi, María Eugenía Perea, Miguel Fernández-Vivas, María Barber, Noemí Llamas, Susana Altaba, Ana Prieto, Vicente Gómez, Mar Martin, Marta Paz, Belen Quesada, Valentí Español, Juan Carlos Montejo, José Manuel Gomez, Gloria Miro, Judith Xirgú, Ana Ortega, Pedro Rascado, Juan María Sánchez, Alfredo Marcos, Ana Tizon, Pablo Monedero, Elisabeth Zabala, Cristina Murcia, Ines Torrejon, Kenneth Planas, José Manuel Añon, Gonzalo Hernandez, María-del-Mar Fernandez, Consuelo Guía, Vanesa Arauzo, José Miguel Perez, Rosa Catalan, Javier Gonzalez, Rosa Poyo, Roser Tomas, Iñaki Saralegui, Jordi Mancebo, Charles Sprung, Rafael Fernández

**Affiliations:** 1 Hospital Sant Joan De Déu, Fundació Althaia Xarxa Universitaria de Manresa, C/ Dr. Joan Soler s. n., 08243 Manresa, Spain; 2 Hospital la Fe de Valencia, Valencia, Spain; 3 Hospital General Yagué de Burgos, Burgos, Spain; 4 Hospital Virgen Arrixaca Murcia, Murcia, Spain; 5 Hospital de Navarra, Pamplona, Spain; 6 Hospital Morales Messeguer, Murcia, Spain; 7 Hospital Universitario de Castellon, Castellon de la Plana, Spain; 8 Hospital Rio Hortega, Valladolid, Spain; 9 Hospital la Moncloa, Madrid, Spain; 10 Hospital Candelaria de Tenerife, Santa Cruz de Tenerife, Spain; 11 Hospital Clínico Universitario de Salamanca, Salamanca, Spain; 12 Fundación Jiménez Díaz, Madrid, Spain; 13 Hospital Central de Asturias, Oviedo, Spain; 14 Hospital Universitario Doce de Octubre, Madrid, Spain; 15 Hospital Gregorio Marañon, Madrid, Spain; 16 Hospital Mataro, Mataro, Spain; 17 Hospital de Granollers, Granollers, Spain; 18 Hospital Montecelo Pontevedra, Pontevedra, Spain; 19 Centro Hospitalario Universitario Santiago Compostela, Santiago de Compostela, Spain; 20 Hospital de la Sant Creu i Sant Pau, Barcelona, Spain; 21 Hospital Virgen de la Concha, Zamora, Spain; 22 Hospital Xeral Cíes Vigo, Vigo, Spain; 23 Clínica Universitaria de Navarra, Pamplona, Spain; 24 Hospital Clínico Universitario de Barcelona, Barcelona, Spain; 25 Hospital Josep Trueta, Girona, Spain; 26 Hospital de Henares, Coslada, Spain; 27 Hospital Moisses Broggi, Sant Joan Despí, Spain; 28 Hospital Virgen de la Luz, Cuenca, Spain; 29 Hospital Infanta Sofía, San Sebastián de los Reyes, Spain; 30 Hospital Mútua de Terrassa, Terrassa, Spain; 31 Hospital Parc Tauli, Sabadell, Spain; 32 Hospital de Terrassa, Terrassa, Spain; 33 Hospital Virgen de las Nieves, Granada, Spain; 34 Hospital General de Vic, Vic, Spain; 35 Hospital Virgen Vega Salamanca, Salamanca, Spain; 36 Hospital Son Llátzer, Palma, Spain; 37 Hospital General de Catalunya, Sant Cugat del Valles, Spain; 38 Hospital de Áraba, Vitoria-Gasteiz, Spain; 39 Hospital de la Santa Creu i Sant Pau, Barcelona, Spain; 40 Hadassh Hebrew University Medical Center, Jerusalem, Israel; 41 Hospital Sant Joan de Deu, Fundació Althaia Xarxa Universitaria de Manresa, Manresa, Spain

**Keywords:** Limitations on life support techniques, Palliative care, Critical care, Intensive care units

## Abstract

**Purpose:**

To determine the frequency of limitations on life support techniques (LLSTs) on admission to intensive care units (ICU), factors associated, and 30-day survival in patients with LLST on ICU admission.

**Methods:**

This prospective observational study included all patients admitted to 39 ICUs in a 45-day period in 2011. We recorded hospitals’ characteristics (availability of intermediate care units, usual availability of ICU beds, and financial model) and patients’ characteristics (demographics, reason for admission, functional status, risk of death, and LLST on ICU admission (withholding/withdrawing; specific techniques affected)). The primary outcome was 30-day survival for patients with LLST on ICU admission. Statistical analysis included multilevel logistic regression models.

**Results:**

We recruited 3042 patients (age 62.5 ± 16.1 years). Most ICUs (94.8%) admitted patients with LLST, but only 238 (7.8% [95% CI 7.0–8.8]) patients had LLST on ICU admission; this group had higher ICU mortality (44.5 vs. 9.4% in patients without LLST; *p* < 0.001). Multilevel logistic regression showed a contextual effect of the hospital in LLST on ICU admission (median OR = 2.30 [95% CI 1.59–2.96]) and identified the following patient-related variables as independent factors associated with LLST on ICU admission: age, reason for admission, risk of death, and functional status. In patients with LLST on ICU admission, 30-day survival was 38% (95% CI 31.7–44.5). Factors associated with survival were age, reason for admission, risk of death, and number of reasons for LLST on ICU admission.

**Conclusions:**

The frequency of ICU admission with LLST is low but probably increasing; nearly one third of these patients survive for ≥ 30 days.

**Electronic supplementary material:**

The online version of this article (10.1186/s40560-018-0283-y) contains supplementary material, which is available to authorized users.

## Background

Decisions to apply limitations on life support techniques (LLSTs) are common in intensive care units (ICUs) worldwide [1–5]. This practice is supported by adequate ethical consensus [1, 6–9] and is even considered an ICU quality indicator [10]. These decisions are usually taken when medical efforts become futile after ICU treatment for some time [11–13].

LLST can entail withholding new treatments, not increasing treatments being applied, or withdrawing treatments. LLSTs are applied in 13 to 34% of all patients admitted to ICUs [14–17] and in 40 to 90% of patients who died in ICUs [7]. LLSTs are associated with high mortality [1, 11, 18]. Lautrette et al. [11] found LLST in 13% of patients (treatments withheld in 39%, not increased in 26%, and withdrawn in 35%); 30-day mortality was 35% in patients in whom the treatment was withheld, 73% in those in whom in the treatment was not increased, and 94% in those in whom the treatment was withdrawn. Another study reported 99% mortality in patients in whom life support was withdrawn and 89% in those in whom further life support measures were withheld during the ICU stay [18].

The Ethicus study [1] of end-of-life practices in European ICUs found that the criteria for deciding LLSTs were patient age, diagnoses, ICU stay, and geographic and religious factors. In a large study, Azoulay et al. [5] found that a higher nurse-to-bed ratio was associated with an increased incidence of LLST, while the availability of an emergency department in the same hospital, full-time presence of intensivists, and presence of physicians during nights and weekends was associated with a decreased incidence of LLST.

In oncologic patients, early LLSTs are mainly related to cancer progression and functional stages; oncological treatment projects and complications leading to ICU admission have a major impact on LLST decisions [19].

In recent years, various authors have proposed that it could be useful to determine LLST on ICU admission for early integrated palliative care [19, 20]. Godfrey et al. [20] reported that 3.2% of patients were admitted to the ICU with orders to withhold life support; half of these survived the ICU stay, and one third were discharged home. More recently, Hart et al. [21] reported that 4.8% of patients had orders to withhold life support before ICU admission.

We aimed to determine the frequency of LLST on ICU admission, associated hospital- and patient-related characteristics, and 30-day survival in patients admitted with LLST. We also explored what types of LLST were applied under what conditions.

## Methods

This prospective observational study included all consecutive patients ≥ 18 years old admitted to 39 ICUs in Spain between 1 May 2011 and 15 June 2011. The ethics committees at each participating center approved the study.

LLST upon ICU admission were defined as orders to withhold or withdraw any life-sustaining treatment. The decisions to apply LLST were performed by a doctor during the guards and by the disciplinary team during the morning hours. Refusal of admission to the ICU was not considered LLST upon ICU admission. Informed consent was requested for the data collection.

We recorded the following characteristics of participating hospitals: number of hospital beds, number of ICU beds, number of step-down/intermediate care beds, funding (public or private), availability of ICU beds, use of restrictive criteria (based on age, previous comorbidity, and/or previous quality of life) for ICU admission, existence of ethics committee guidelines for LLST, and whether coronary and/or stroke patients were admitted.

At admission to the ICU, we recorded patients’ age, sex, reason for admission, prior functional status (Knaus chronic health status score), and risk of death according to severity scales. We also recorded decisions to apply LLST (withhold new treatment, not increase current treatment, or withdraw treatment), the specific life support techniques to be limited (cardiopulmonary resuscitation, endotracheal intubation, noninvasive ventilation, vasopressor drugs, dialysis, and/or transfusion of blood products), and reason for LLST decision (age, severe chronic disease, prior functional limitations, unacceptable quality of life despite possible recovery from the acute process, advanced life directives, no expectation of surviving the hospital stay, and anticipated irreversibility of the current process within 24 h). We also recorded the reversal of LLST orders during the ICU stay.

On ICU discharge, we evaluated patients’ clinical status and prognosis with the Sabadell score [22], which classifies patients into five groups: SS0 = good prognosis, SS1 = poor long-term (> 6 months) prognosis with no limits on ICU readmission, SS2 = poor short-term prognosis (< 6 months) with debatable ICU readmission, SS3 = death expected during hospitalization with ICU readmission not applicable, and SS4 = death in ICU.

The primary outcome was 30-day survival for patients with LLST on ICU admission. Secondary outcomes were decisions to withhold vs. withdraw life support at ICU admission, ICU length of stay, Sabadell score at ICU discharge, and in-hospital mortality.

### Statistical analysis

We summarize categorical variables as absolute and relative frequencies and continuous variables as means and standard deviations or medians and interquartile ranges. To compare patients with vs. without LLST, we used Student’s *t* tests for normally distributed continuous variables, nonparametric Mann-Whitney tests for non-normally distributed continuous variables, and chi-square tests, Fisher’s exact tests, or the Monte Carlo method (in 2 × 2 contingency tables or nx2 when expected frequencies < 5) for categorical variables.

To determine whether the contextual effect of the hospital was related to LLST, we used a random-effects multilevel logistic regression model with the hospital as a second-level variable (random effect) and the patient and center characteristics that were associated with LLST in the bivariate analysis as first-level variables. We used odds ratios and median odds ratios (MOR) to measure the association between each covariate and LLST [23]. The MOR is a measure of the variation between the rates of LLST at different hospitals that is unexplained by the modeled risk factors; it is defined as the median of the set of odds ratios that could be obtained by comparing two patients with identical patient-level characteristics from two randomly chosen hospitals.

Survival was analyzed by Kaplan-Meier curves and compared by log-rank test. Crude and adjusted hazard ratios and 95% confidence intervals (CI) were calculated using Cox proportional regression models. We introduced covariates with *p* ≤ 0.20 in the bivariate analysis or with evidence of an association in the literature into the multivariate regression model, using a researcher-controlled backward exclusion strategy. Proportionality of hazards was verified by examining Schoenfeld residual plots.

All tests were two-sided, and the significance was set at *p* < 0.05. For statistical analyses, we used IBM® SPSS® Statistics for Windows v.20 and Stata® v.10.

## Results

Of the 39 ICUs, 34 (87.2%) received public funding; the median number of hospital beds was 575 (360–800) and the median number of ICU beds was 17 (11–22). Beds were often available in 33 (84.6%) ICUs; 33 (84.6%) had restrictive admission policies, 37 (94.9%) had clinical ethics committees, and 13 (33%) had guidelines for LLST. Intermediate care units were available in 13 (33.3%) centers. (Additional file 1: Table S1).

During the study period, participating ICUs admitted 3042 patients (age, 62.5 ± 16.1 years; 1935 (63.6%) men). The reason for ICU admission was worsening of chronic disease in 353 (11.6%), coma/encephalopathy in 386 (12.7%), and sepsis in 411 (13.5%). The Knaus chronic health status score classified patients’ prior functional status as class A in 57.4%, class B in 31.0%, class C in 9.4%, and class D in 2.1%. The median risk of death predicted by the severity scales was 14% [5.8–32.9%] (Additional file 1: Table S2).

Most ICUs (94.8%) accepted patients with LLST at ICU admission. A total of 238 (7.8%) [95% CI 7.0–8.8%] patients (age, 73.0 ± 13.5 years; 130 (55%) men) had LLST on ICU admission, with a median predicted risk of death of 46.3% [24.0–63.9%].

Reasons for LLST were severe chronic disease (*n* = 143; 60.1%), prior functional limitations (*n* = 110; 46.2%), advanced age (*n* = 90; 37.8%), null expected survival (*n* = 83; 34.9%), unacceptable quality of life (*n* = 63; 26.5%), irreversibility within 24 h (*n* = 50; 21.0%), advanced life directives (*n* = 12; 5.0%), and others (*n* = 15; 6.3%); there were 2.4 ± 1.1 reasons recorded per patient (Additional file 1: Table S3).

Table 1 reports the life support techniques limited and the type of limitation on each. The most common type of LLST on ICU admission was withholding, especially for invasive treatments. Withholding or withdrawing is most commonly referred to invasive measures (cardiopulmonary resuscitation maneuvers, dialysis, and intubation). Decisions to limit noninvasive life support measures (vasoactive drugs, noninvasive ventilation, and transfusions) were less frequent and nearly always involved withholding rather than withdrawing treatment. Withdrawing life invasive support was very uncommon. LLST orders were reversed only in seven (2.9%) patients.

**Table 1:** Life support techniques limited in patients with orders to limit life support on admission to the intensive care unit

		Type of limitation	
Technique		Withhold	Withdraw
Invasive life support			
Cardiopulmonary resuscitation	215 (91.5%)	215 (91.5%)	0 (0%)
Dialysis	209 (89.3%)	203 (86.8%)	6 (2.6%)
Intubation	147 (63.9%)	126 (54.8%)	21 (9.1%)
Noninvasive life support			
Vasopressors	104 (45.0%)	96 (41.5%)	8 (3.5%)
Noninvasive ventilation	61 (26.9%)	60 (26.4%)	1 (0.4%)
Blood transfusions	59 (25.5%)	55 (23.8%)	4 (1.7%)

In the bivariate analysis, LLSTs were associated with older age (73.0 vs. 61.6 years; *p* < 0.001) and female sex (9.6% in women vs. 6.8% in men; *p* = 0.006) (Additional file 1: Tables S3 and S4). The most common reasons for ICU admission in patients with LLST were worsening of chronic disease (19.0%) and coma/encephalopathy (15.8%). Compared to patients without LLST, patients with LLST had a higher risk of death (46.3 vs. 12.0%; *p* < 0.001).Worsening prior functional status was associated with more LLST (from 46.0% in class D down to 2.1% in class A).

Table 2 shows the patient and hospital characteristics independently associated with LLST on ICU admission. Multilevel logistic regression found a contextual effect of hospital on LLST decisions on ICU admission (MOR = 2.30; model A; Table 2). Hospital characteristics associated with LLST were the lack of intermediate care units and the incapability to treat severely ill patients with LLST outside the ICU (model B; Table 2). Patient characteristics independently associated with LLST were age; admission for coma, encephalopathy, or worsening of chronic disease; risk of death; and prior functional status class B, C, or D (Additional file 1: Table S5).

**Table 2:** Associations between limitations on life support on ICU admission and patient and hospital characteristics. Adjusted odds ratio (aOR) and 95% confidence interval (95% CI)

	Limitations, *n* = 238	No limitations, *n* = 2804	Model A aOR (95% CI)	Model B aOR (95% CI)
Patient characteristics				
Age	73.0 ± 13.5	61.6 ± 16.0	1.04 (1.03–1.06)	1.05 (1.03–1.06)
Female sex	106 (44.5%)	1000 (35.6%)	1.30 (0.93–1.81)	1.30 (0.92–1.78)
Reason for ICU admission				
Other Sepsis Coma or encephalopathy Worsening of chronic disease	77 (32.3%) 33 (13.9%) 61 (25.6%) 67 (28.1%)	1816 (64.8%) 377 (13.4%) 326 (11.6%) 285 (10.2%)	1 0.94 (0.57–1.57) 3.96 (2.50–6.30) 2.34 (1.50–3.66)	1 0.97 (0.58–1.62) 3.88 (2.45–6.13) 2.34 (1.50–3.66)
Predicted risk of death (%)	46.3 [24.0–63.9]	12.0 [5.1–29.0]	1.03 (1.02–1.04)	1.03 (1.02–1.04)
Prior functional Knaus status:				
Class A Class B Class C Class D	37 (15.5%) 96 (40.3%) 76 (31.9%) 29 (12.2%)	1708 (60.9%) 848 (30.2%) 211 (7.5%) 34 (1.2%)	1 3.80 (2.44–5.92) 13.44 (8.00–22.58) 36.94 (17.34–78.71)	1 3.71 (2.39–5.77) 13.30 (7.93–22.32) 36.77 (17.29–78.20)
Hospital characteristics				
Intermediate care unit available				
Yes No	43 (18.1%) 195 (81.9%)	826 (29.4%) 1978 (70.5%)		1 1.85 (1.00–3.44)
Patients with limitations on life support outside the ICU				
Yes No	143 (60.1%) 95 (39.9%)	1928 (68.7%) 876 (31.2%)		1 2.57 (1.45–4.57)
Hospital variance (SE) LR test; *p* value			0.765 (0.271) 54.38; *p* < 0.001	0.453 (0.191) 24.69; *p* < 0.001
Intraclass correlation coefficient			0.189	0.121
Median odds ratio (95% CI)			2.30 (1.59–2.96)	1.90 (1.31–2.38)

Mean ± standard deviation; *n* (row %); median [interquartile range]; *SE* standard error

Model A—random effects multilevel logistic regression model with hospital as a second-level variable (random effect) and the patient characteristics as first-level variables

Model B—random effects multilevel logistic regression model with hospital as a second-level variable (random effect) and the patient and center characteristics as first-level variables

Median ICU stay was not different between patients with or without LLST at ICU admission. In LLST patients, the most common Sabadell score at ICU discharge was SS4 (death, 44.5%), followed by SS2 (poor short-term prognosis, 29.8%) and SS3 (expected survival null, 11.3%), while in patients without limitations, the most common was SS0 (good prognosis, 64.0%) (Table 3).

**Table 3:** Clinical outcome according to limitations on life support on ICU admission

	Limitations, *n* = 238	No limitations, *n* = 2804	*p* value
ICU length of stay, days	3 [1–6]	3 [1–6]	0.711^a^
Sabadell score at ICU discharge:			
SS0—good prognosis SS1—poor long-term prognosis SS2—poor short-term prognosis SS3—expected to die SS4—died	7 (2.9%) 27 (11.3%) 71 (29.8%) 27 (11.3%) 106 (44.5%)	1794 (64.0%) 525 (18.7%) 182 (6.5%) 37 (1.3%) 264 (9.4%)	< 0.001^b^
ICU mortality Ward mortality Hospital mortality	106 (44.5%) 35 (14.7%) 141 (59.2%)	264 (9.4%) 84 (3.1%) 348 (12.7%)	< 0.001^b^ < 0.001^b^ < 0.001^b^

Median [interquartile range]; *n* (% of column)

^a^Mann-Whitney *U*

^b^Pearson chi-square

**Fig. 1:**
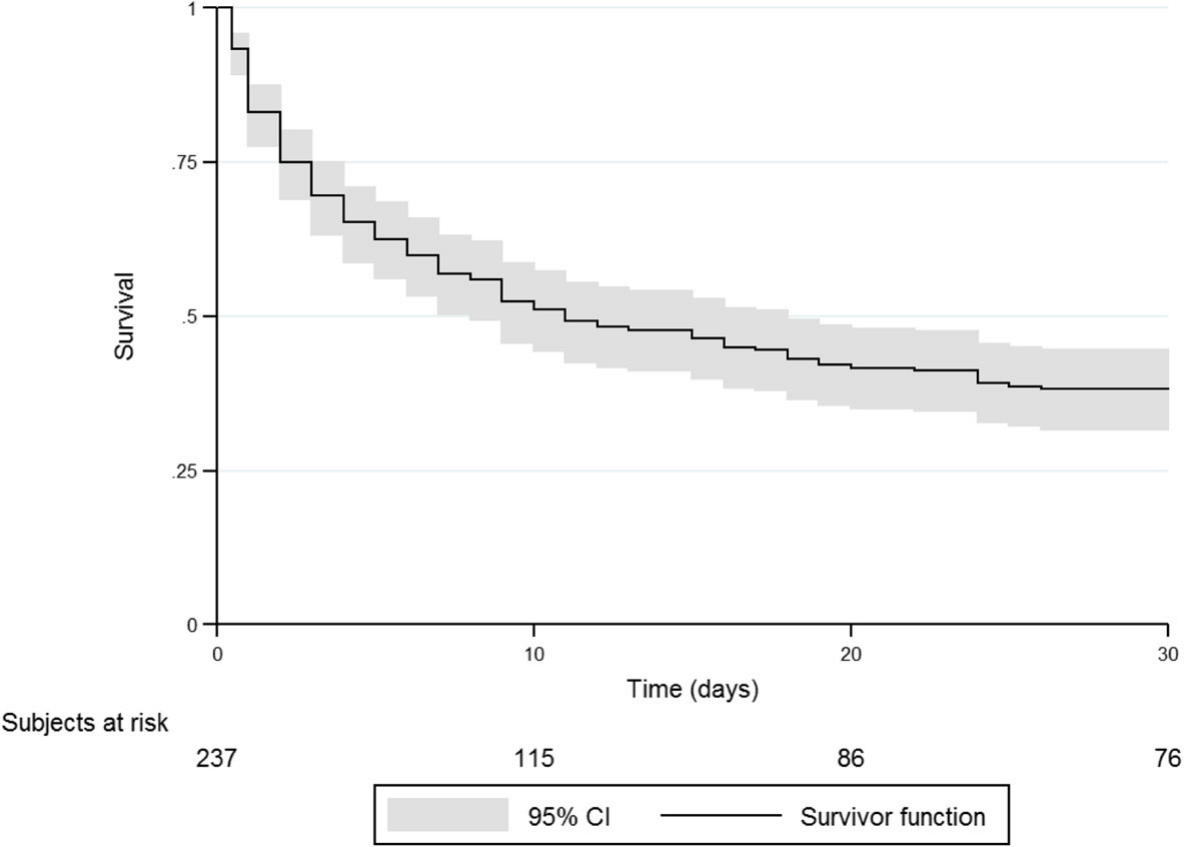
Thirty-day overall survival function

A greater proportion of patients with LLST on ICU admission died in the ICU (44.5 vs. 9.4% in those without; *p* < 0.001). Hospital mortality was higher in patients with LLST (59.2 vs. 12.7% in those without; *p* < 0.001) (Fig. 1).

Thirty-day survival in patients with LLST at ICU admission was 38% (95% CI 31.7–44.5). Independent predictors of worse 30-day survival were older age, ICU admission for coma/encephalopathy or sepsis, higher predicted risk of death, and more reasons for LLST decision (Table 4).

**Table 4:** Predictive factors for 30-day survival in patients with limitations on life support at ICU admission

	Died, *n* = 141	Survived, *n* = 97	Unadjusted HR (95% CI)	Adjusted HR (95% CI)
Age, year	71.4 ± 12.2	75.2 ± 15.0	0.99 (0.98–0.99)	0.98 (0.97–0.99)
Female sex	58 (54.7%)	48 (45.3%)	1.16 (0.83–1.62)	1.2 (0.85–1.71)
Reason for ICU admission				
Worsening of chronic disease	31 (46.3%)	36 (53.7%)	1	1
Coma or encephalopathy	48 (78.7%)	13 (21.3%)	2.51 (1.59–3.95)	1.79 (1.10–2.91)
Sepsis	22 (66.7%)	11 (33.3%)	1.80 (1.04–3.11)	1.64 (0.90–2.97)
Other	40 (51.9%)	37 (48.1%)	1.23 (0.77–1.97)	1.23 (0.68–1.97)
Predicted risk of death, %	54.6 [38.9–72.0]	24.0 [12.0–45.0]	1.03 (1.02–1.03)	1.02 (1.02–1.03)
Prior functional Knaus status				
Class A	26 (70.3%)	11 (29.7%)	1	1
Class B	55 (57.3%)	41 (42.7%)	0.78 (0.49–1.25)	0.83 (0.49–1.38)
Class C	45 (59.2%)	31 (40.8%)	0.83 (0.51–1.34)	0.56 (0.31–1.02)
Class D	15 (51.7%)	14 (48.3%)	0.71 (0.38–1.35)	0.45 (0.22–0.92)
Criteria for limitations, number	2 [2–3]	2 [1–3]	1.33 (1.14–1.55)	1.45 (1.22–1.76)
Limited techniques, number	3 [2–5]	3 [2–4]	1.16 (1.03–1.31)	1.10 (0.97–1.25)

Mean (SD); *n* (% of the row); median [interquartile range]

Survival differed in function of whether both invasive and noninvasive or only noninvasive life support techniques were limited. Withholding and withdrawing noninvasive measures were both associated with lower survival. Not increasing the dose of vasoactive drugs was associated with greater survival than withdrawing them. Withholding invasive measures was associated with greater survival than withdrawing them, with the exception of intubation. Additional file 1: Figure S1 reports the survival related to the life support technique limited.

## Discussion

To our knowledge, this is the first prospective multicenter study to evaluate the clinical, structural, and demographic factors associated with LLST decisions at ICU admission and their associations with survival.

Most centers admitted patients with LLST orders, and those patients accounted for 7.8% of all ICU admissions. This rate is somewhat higher than previously reported and may be influenced by the wide availability of ICU beds in the study period. Only two centers refused to admit LLST patients, and both had 100% occupancy rate. These two centers admitted more severe patients (median risk of death 40 vs. 22% in the other centers) and had step-down units (compared with only 3 3% of the other centers), so patients with LLST in these two centers were probably admitted to step-down units.

Although LLST patients were sicker, their 30-day survival was 38%. LLST at ICU admission were related to patient factors (age, comorbidities, functional status, and predicted risk of death) and hospital factors. Survival was affected by the same patient-related factors and by the number of reasons for LLST, the type of limitation, and the specific life support techniques limited. A study in Brazil found 9.8% of patients admitted to the ICU had limitations on advanced life support, and LLST decisions were associated with older age, clinical diagnosis, Karnofsky performance status score < 40%, and SAPS3 score > 49 points [24]. As in our study, Godfrey et al. [20] found that older age, more comorbid disease, and more acute physiological disturbance were associated with mortality in patients with LLST at admission; moreover, 30% were discharged directly to their homes.

Our findings suggest a trend toward an increase in LLST at ICU admission; whereas earlier studies found limitations in only 1 to 3% of patients [20, 25], we found limitations in nearly 8%. This trend may be related to patients’ increasing age and complexity (associated comorbidities and frailty) due to increased life expectancy, changes in patterns of end-of-life trajectories [26], changes in ICU admission criteria, and advanced life directives in patients with advanced cancer and organ failure [24]. Thus, many patients (oncological, hematologic, and geriatric patients) are admitted to ICUs for therapeutic tests or conditioning treatments because ICU admission seems to improve outcomes in these situations; for example, in cancer patients requiring mechanical ventilation are widely viewed as poor candidates for intensive care unit (ICU) admission. One study of patients has demonstrated that if patients were admitted at the earliest phase of the malignancy (diagnosis < 30 days) without any restriction, the survival was 40% in mechanically ventilated cancer patients who survived to day 5 and 21.8% overall. All patients were prospectively included in The ICU Trial, consisting of a full-code ICU admission followed by reappraisal of the level of care on day 5. It would be interesting to conduct efficiency studies in these case [27–30]. Nevertheless, although the frequency of LLST at ICU admission is increasing, it is still lower than the frequency of limitations applied during the ICU stay (13–34%) [3, 17].

In the univariate analysis, hospital mortality was associated with the reason for admission, previous poor functional status, LLST at admission, age, and risk of death. In the multivariate analysis, hospital mortality was associated with age, reason for admission type II/III, predicted risk of death, poor functional status (C and D), and LLST at admission.

Decision-making about LLST is affected by patient-related factors and factors related to health professionals [1]. Pathophysiological factors often preclude ICU patients from making decisions, and the burden of decision-making falls on their relatives or legal representatives. However, up to half of ICU patients’ relatives do not want to participate in the decision-making about LLST [5]. Trends toward patient empowerment in the near future through more active participation and shared decision-making models will likely influence decisions to limit life support before ICU admission [31, 32]. Factors related to professionals also affect LLST decisions and account for some of the variability in decision-making [33].

On average, more than two reasons were given for LLST decisions; the most common were directly related to preexisting chronic disease and prior functional status, whereas poor quality of life was rarely considered. We found that LLSTs were more prevalent in patients admitted for worsening of severe chronic disease, as suggested by Godfrey et al. [20], who reported that chronic disease was the reason LLST in 77%. Prognosticating in these patients is more straightforward, so it is easier to establish LLST in advance.

We do not know if the decisions were proposed by the patient, the family, or unilaterally by the doctor on duty or the medical team (that it could be a point of interest for future research), but all the decisions were endorsed later in the clinical session.

We found that LLST decisions at ICU admission usually entailed withholding invasive life support measures. By contrast, LLST decisions made during the ICU stay more often involved withdrawing measures when they proved futile [34]. This is probably due to the different profiles of patients admitted with LLST and those without. The EPIPUSE study [35] showed that patients with LLST decided during the ICU stay were younger and rarely admitted for worsening of chronic disease or coma/encephalopathy; the risk of death predicted by severity scores in the EPIPUSE study was also notably lower than in our study [36].

The life support measure most often limited to ICU admission was cardiopulmonary resuscitation, commonly associated with the decision not to increase life support [37]. These findings also differ from LLST decided during the ICU stay, where most decisions are not to increase life support or to withdraw life support after they prove futile [3].

At ICU discharge, patients admitted with LLST had a worse prognosis, and the mortality in this group was approximately fourfold that of patients admitted without LLST, unsurprisingly, given the difference in severity at ICU admission [38].

In line with Godfrey et al. [20], ICU and in-hospital mortality were higher in patients with LLST at ICU admission in our study, due to both LLST and greater severity. Nevertheless, our 30-day survival in patients with LLST on ICU admission was 38%, slightly higher than the 30% previously reported [20].

Interestingly, mortality is lower (up to 90%) when LLSTs are decided on ICU admission than when decided during the ICU stay [11], most likely because patients in the latter group are sicker and because LLSTs are decided after therapy failure and more often entail withdrawing rather than withholding life support. Furthermore, survival differed with the type of LLST at admission; survival was greater in patients in whom life support measures were withheld than in those in whom they were withdrawn similar to what happens when the decision is taken during the ICU stay [11, 15].

Regarding survival curves according to the type of limited life support, two limitations must be taken into account for correct reading; one is that we do not know the patient’s previous starting point (we do not know if he was intubated on admission before making decisions), and the second is that for each patient with decisions could be marked several limited life supports at once but the analysis has been done in isolation for each life support without taking into account their interrelation. Therefore, the results should always be interpreted with caution.

It seems paradoxical that life support intubation has better survival in the form not start than the nonlimitation and withdrawal when the other vital supports are the opposite. An explanation could lie in the fact that some patients are intubated at home by the urgent prehospital care and that after arriving at the hospital and before being admitted to the ICU they are cataloged as LLST.

The factors independently associated with LLST decisions at ICU admission in our study agree with those reported in other studies [20], although the contextual effect of hospital on LLST decisions in our study is new. The factors independently associated with survival in patients with LLST at ICU admission were related to the underlying chronic disease, better prior functional status, less severe disease, and fewer reasons for the LLST decision.

Nevertheless, further studies are needed to define how these decisions are made at admission, and how this affects patients’ outcomes, about the validation of tools to quantify frailty and about comorbidity and performance status [39]. Furthermore, studies should assess survivors’ quality of life and satisfaction to evaluate the efficiency of ICU admission in these patients.

### Limitations of the study

Not including patients with LLST decided outside the ICU and therefore not admitted may have underestimated the global incidence of LLST. Our sample (about six patients with LLST on ICU admission per center) precluded an analysis of the contextual effect of hospital on survival, although a systematic review found LLST varied among countries and regions [40]. Finally, we cannot rule out a seasonal bias, as the study took place during a 6-week period in late spring, but LLST has never been associated with seasonal bias.

## Conclusions

Most ICUs admitted patients with LLST. The frequency of such admissions is low but probably increasing; nearly one third of these patients survive for 30 days. The main factors associated with LLST decisions at ICU admission and with 30-day survival in patients with these limitations are age, reason for ICU admission, and predicted risk of death.

### Additional file

Additional file 1: **Table S1.** Hospital characteristics. **Table S2.** Patient characteristics. **Table S3.** Reasons for limitations on life support at admission to the ICU. **Table S4.** Bivariate analysis. Patient characteristics associated with LLST. Crude odds ratio (OR) and 95% confidence interval. **Table S5.** Bivariate analysis. Hospital characteristics associated with LLST. Crude odds ratio (OR) and 95% confidence interval. **Figure S1.** Thirty-day overall survival function according to the specific support measures limited and the type of limitation. (RTF 56201 kb)

## Appendix

### LLST investigators and centers

ANDALUCIA: José Miguel Pérez-Villares (Hospital Virgen Nieves de Granada). ASTURIAS: Valentín Español-Boren (Hospital Universitario central de Asturias). ISLAS BALEARES: Rosa Poyo-Guerrero (Hospital Son Llatzer). CANARIAS: Mar Martín-Velasco (Hospital Candelaria Tenerife). CASTILLA Y LEON: María Eugenia Perea-Rodríguez (Complejo Asistencial Universitario de Burgos. Hospital General Yagué), Alfredo Marcos-Gutiérrez (Hospital Virgen de la Concha de Zamora), Mercedes Lara-Calvo (Hospital Rio Hortega de Valladolid), José Manuel Añon-Elizalde (Hospital Virgen de la Luz de Cuenca), Marta Paz-Pérez (Hospital Clínico Universitario de Salamanca). CATALUÑA: Gloria Miro-Andreu (Hospital de Mataró), Juan María Sánchez-Segura (Hospital de la Santa Creu i Sant Pau de Barcelona), Elisabeth Zabala-Jiménez (Hospital Clínico Barcelona UCI quirúrgica), Roser Tomas-Puig (Hospital General Cataluña en Sant Cugat), Cristina Murcia-Gubianes (Hospital de Girona), Kenneth Planas (Hospital de Sant Joan Despí Moises Broggi), Judith Xirgú-Cortacans (Hospital Universitario de Granollers), Mar Fernández-Fernández (Hospital Universitario Mútua de Terrassa), Consuelo Guía-Rambla (Hospital de Sabadell), Vanesa Arauzo-Rojo (Hospital de Terrassa), Rosa Catalan-Ibars (Hospital Universitari de Vic), Javier Gónzalez-Robledo (Hospital Virgen de la Vega de Salamanca). GALICIA: Ana Ortega-Montes (Hospital Montecelo de Pontevedra), Pedro Rascado-Sedes (CHU Santiago Compostela), Ana Tizón-Varela (Complejo Hospital Xeral Cíes de Vigo), Pedro Rascado-Sedes (Hospital Santiago de Compostela). COMUNIDAD DE MADRID: Juan Carlos Montejo-González (Hospital 12 de octubre), José Manuel Gómez-García (Hospital Gregorio Marañón), Inés Torrejón-Pérez (Hospital de Henares), Gonzalo Hernández-González (Hospital Infanta Sofía). REGIÓN DE MURCIA: Noemí Llamas-Fernández (Hospital Morales Meseguer de Murcia), Miguel Fernández-Vivas (Hospital Virgen de la Arrixaca de Murcia). COMUNIDAD FORAL DE NAVARRA: Pablo Monedero-Rodríguez (Clínica Universitaria de Navarra), María Barber-Ansón (Hospital de Navarra), Belén Quesada-Bellber (Fundación Jiménez Díaz), Vicente Gómez-Tello (Hospital de la Moncloa). PAIS VASCO: Iñaki Saralegui-Reta (Hospital Santiago Apóstol de Álava). COMUNIDAD VALENCIANA: Susana Altaba-Tena (Hospital General de Castellón), Begoña Balerdi-Pérez (Hospital la Fe de Valencia).

## Abbreviations

ICU: Intensive care unit
LLST: Limitations on life support technique
MOR: Median odds ratio
SS: Sabadell score
