# Supplementary material for: Limitation of life support techniques at admission to the intensive care unit: a multicenter prospective cohort study
Source: J Intensive Care. 2018 Apr 13;6:24. doi: 10.1186/s40560-018-0283-y (PMC5899386; doi:10.1186/s40560-018-0283-y)
Supplement: Supplementary file 1 — Table S1. Hospital characteristics. Table S2. Patient characteristics. Table S3. Reasons for limitations on life support at admission to the ICU. Table S4. Bivariate analysis. Patient characteristics associated with LLST. Crude odds ratio (OR) and 95% confidence interval. Table S5. Bivariate analysis. Hospital characteristics associated with LLST. Crude odds ratio (OR) and 95% confidence interval. Figure S1. Thirty-day overall survival function according to the specific support measures limited and the type of limitation. (RTF 56201 kb) [file 40560_2018_283_MOESM1_ESM.rtf]

Supplementary table 1.Table S1 Hospital characteristics.

N=39	
Public financing model	34 (87.2%)	
Number of hospital beds	575 [360-800]	
Number of ICU beds	17 [11-22]	
Step-down/intermediate care	13 (33.3%)                                                          	
Number of step-down/intermediate care beds	6 [4-15]	
Usual availability of ICU beds	33 (84.6%)                                                                    	
Restrictive criteria for admission to the ICU	33 (84.7%)	
Admitted patients who had limitations on life support	37 (94.9%)	
Guidelines for the limitation of life support 	13 (33.3%)	
Clinical ethics committee	37 (94.9%)	
Admitted coronary patients and/or stroke patients	35 (89.7%)	
N (% Yes); Median [interquartile range]


Supplementary table 2.Table S2 Patient characteristics.


N=3042	
Age	62.5 (16.1)	
Sex		
Men	1935 (63.6%)	
Women	1106 (36.4%)	
Reason for ICU admission		
Worsening of chronic disease          	352 (11.6%)	
Coma or encephalopathy                                       	387 (12.7%)	
Sepsis        	           410 (13.5%)	
Other                                                                                                                             	1893 (62.2%)	
Severity scale                                                                         		
SAPS II	157 (5.2%)	
SAPS 3	1174 (38.9%)	
APACHE II                                                   	1687 (55.9%)	
Associated risk of death in severity scales (%)                                              	14 [5.8-32.9]                                                                    	
Prior functional status (Knaus)		
Class A                                                            	1745 (57.4%)	
Class B                                                            	944 (31.0%)	
Class C	287 (9.4%)	
Class D	63 (2.1%)	
Limitations on life support on ICU admission	238 (7.8%)	
Media (SD); N (%); Median [interquartile range]


Supplementary table 3.Table S3 Reasons for limitations on life support at admission to the ICU.


N=238	
Severe chronic disease	143 (60.1%)	
Prior functional limitations	110 (46.2%)	
Age	90 (37.8%)	
Null probability of surviving the hospital stay	  83 (34.9%)	
Qualitative futility	63 (26.5%)	
Irreversibility of the current process in the first 24 hours	50 (21.0%)	
Specifications in the patient's advanced life directive	12 (5.0%)	
Other	15 (6.3%)	


Supplementary table 4.Table S4 Bivariate analysis. Patient characteristics associated with LLST. Crude odds ratio (OR) and 95% confidence interval.

	LLST
n=238	No LLST
n=2804	p-value	Crude OR	95% CI	
Age	73.0 (13.5)	61.6 (16.0)	<0.001a	1.06	1.05-1.07	
Sex						
Men	132 (6.8%)	1803 (93.2%)	0.006b	1		
Women                               	106 (9.6%)                                   	1000 (90.4%)		1.45                                   	1.11-1.89	
Reason for ICU admission						
Worsening of chronic disease          	67 (19.0%)	285 (81.0%)	<0.001b	1		
Coma or encephalopathy                                       	61 (15.8%)	326 (84.2%)		2.06	1.35-3.15	
Sepsis        	33 (8.0%)	377 (92.0%)		4.41	3.09-6.30	
Other                                                                                                                             	77 (4.1%)	1816 (95.9%)		5.54	3.91-7.87	
Associated risk of death in severity scales (%)                                              	46.3 [24.0-63.9]	12.0 [5.1-29.0]	<0.001a	1.04	1.03-1.04	
Prior functional status (Knaus)						
Class A                                                            	37 (2.1%)	1708 (97.9%)	
<0.001b	1		
Class B                                                            	96 (10.2%)	848 (89.8%)		5.23	3.54-7.70	
Class C	76 (26.5%)	211 (73.5%)		16.63	10.95-25.26	
Class D	29 (46.0%)	34 (54.0%)		39.37	21.76-71.24	

Media (SD); N (row %); Median [interquartile range] 
a Mann-Whitney test, b Chi-squared test


Supplementary table 5.Table S5 Bivariate analysis. Hospital characteristics associated with LLST. Crude odds ratio (OR) and 95% confidence interval.

	LLST
n=238	No LLST
n=2804	p-value	Crude OR	95% CI	
Financing model						
Private              	22 (4.5%)	470 (95.5%)	0.002b	1		
Public	216 (8.5%)	2334 (91.5%)		1.98	1,26-3,10	
Number of hospital beds						
First quartile	56 (7.8%)	664 (92.2%)	0.976b	1		
Second quartile                                         	67 (8.0%)	766 (92.0%)		1.04	0.72-1.50	
Third quartile	54 (8.0%)	621 (92.0%)		1.03	0.70-1.52	
Fourth quartile                     	61 (7.5%)	753 (92.5%)		0.96	0.66-1.40	
Number of ICU beds						
First quartile	69 (9.1%)	691 (90.9%)	0.162b	1		
Second quartile                                         	36 (6.2%)	541 (93.8%)		0.67	0.44-1.01	
Third quartile	62 (7.0%)	819 (93.0%)		0.76	0.53-1.08	
Fourth quartile                     	71 (8.6%)	753 (91.4%)		0.94	0.67-1.34	
Step-down/intermediate care						
Yes	43 (4.9%)	826 (95.1%)	<0.001b	1		
No	195 (9.0%)	1978 (91.0%)		1.89	1.35-2.66	
Usual availability of ICU beds						
Yes	196 (7.7%)	2355 (92.3%)	0.511b	1		
No	42 (8.6%)	449 (91.4%)		1.12	0.79-1.59	
Restrictive criteria for admission to the ICU						
No	26 (5.4%)	452 (94.6%)	0.034b	1		
Yes	212 (8.3%)	2352 (91.7%) 		1.57	1.03-2.38	
Guidelines for the limitation of life support						
Yes	70 (6.4%)	1028 (93.6%)		1		
No	168 (8.6%)	1776 (91.4%)	0.025b	1.40	1.04-1.86	
Clinical ethics committee						
No	7 (4.4%)	151 (95.6%)	0.103b	1		
Yes	231 (8.0%)	2653 (92.0%)		0.53	0.25-1.15	


Admitted patients who had limitations on life support						
Yes	143 (6.9%)	1928 (93.1%)		1		
No	95 (9.8%)	876 (90.2%)	0.006b	1.46	1.11-1.92	
Admitted coronary patients and/or stroke patients						
No	19 (6.3%)	284 (93.7%)	0.289b	1		
Yes	219 (8.0%)	2520 (92.0%)		1.30	0.80-2.11	

N (row %) 
b Chi-squared test


Supplementary figure 1.Figure S1 Thirty-day overall survival function according the specific support measures limited and the type of limitation.

INVASIVE LIFE SUPPORT 


NONINVASIVE LIFE SUPPORT
